# Robotic Salvage Lymph Node Dissection After Radical Prostatectomy

**DOI:** 10.1590/S1677-5538.IBJU.2014.0614

**Published:** 2015

**Authors:** Fabio C. M. Torricelli, Arnaldo Cividanes, Giuliano B. Guglielmetti, Rafael F. Coelho

**Affiliations:** 1 Hospital das Clínicas da Faculdade de Medicina da Universidade de São Paulo, SP, Brasil; 2 Hospital 9 de Julho, SP. Brasil; 3 Instituto do Câncer do Estado de São Paulo (ICESP), SP, Brasil

## Abstract

**Introduction and objective:**

Radical prostatectomy is a first-line treatment for localized prostate cancer. However, in some cases, biochemical recurrence associated with imaging-detected nodal metastases may happen. Herein, we aim to present the surgical technique for salvage lymph node dissection after radical prostatectomy.

**Materials and Methods:**

A 70 year-old asymptomatic man presented with a prostate-specific antigen (PSA) of 7.45ng/ mL. Digital rectal examination was normal and trans-rectal prostate biopsy revealed a prostate adenocarcinoma Gleason 7 (3+4). Pre-operative computed tomography scan and bone scintigraphy showed no metastatic disease. In other service, the patient underwent a robotic-assisted radical prostatectomy plus obturador lymphadenectomy. Pathologic examination showed a pT3aN0 tumor. After 6 months of follow-up, serum PSA was 1.45ng/mL. Further investigation with 11C–Choline PET/CT revealed only a 2-cm lymph node close to the left internal iliac artery. The patient was counseled for salvage lymph node dissection.

**Results:**

Salvage lymph node dissection was uneventfully performed. Operative time was 1.5 hour, blood loss was minimal, and there were no intra- or postoperative complications. The patient was discharged from hospital in the 1st postoperative day. After 12 months of follow-up, his PSA was undetectable with no other adjuvant therapy.

**Conclusion:**

Robotic salvage pelvic lymph node dissection is an effective option for treatment of patients with biochemical recurrence after radical prostatectomy and only pelvic lymph node metastasis detected by C11-Choline PET/CT.

## ARTICLE INFO

**ARTICLE INFO Available at:** **www.brazjurol.com.br/videos/july_august_2015/Torricelli_819_820video.htm**

**Int Braz J Urol. 2015; 41 (Video #7): 819-20**
