# Supplementary material for: Robotic Salvage Lymph Node Dissection After Radical Prostatectomy
Source: Int Braz J Urol. 2015 Jul-Aug;41(4):819–20. doi: 10.1590/S1677-5538.IBJU.2014.0614 (PMC4757015; doi:10.1590/S1677-5538.IBJU.2014.0614)
Supplement: Related texts

# Letter: EDITORIAL COMMENT

**Authors:** Vipul Patel, Vladimir Mouraviev

**Affiliations:** Director of Urologic Oncology, Florida Hospital Cancer Institute, FL, USA. E-mail: vipul.patel.md@flhosp.org

To date, an implementation of novel imaging techniques, such as PSMA or 11C-choline PET fused with CT or MRI, and diffusion-weighted MRI imaging (DWI), seem to significantly improve restaging of lymph node (LN) status after radical prostatectomy. From physician and patient prospects, it is outmost of importance to accurately detect the solitary LNs considering them as a final local resort of residual cancer disease. From this point, the precise preoperative mapping of potential targets can be a prerequisite for successful salvage robotic-assisted radical prostatectomy (sRARP). The recent data suggested that sLND can delay clinical progression and postpone hormonal therapy in almost one-third of the patients, although the majority of them will have biochemical failure (1).

The presented videocase demonstrated that 11C-choline PET/CT scan may be useful as a reliable and promising tool for restaging of LN status in patients with biochemical recurrence of disease after RARP. However, more evidence-based data are needed to prove further validation of accuracy of this scan especially with low PSA threshold (less than 0.5-1.0ng/mL). Furthermore, the contemporary literature data and our initial clinical experience show a value of other radiopharmaceutical agent such as PSMA PET/CT scan as even more precise diagnostic modality. Otherwise, new data with DW-MRI use also seem to have favorable performance characteristics as well. For instance, Elbert et al. (3) examined the results on 29 patients with DW-MRI using quantitative parameter such as ADC (apparent diffusion coefficient) cut-off of 1.30x10-3 reporting a sensitivity, specificity and discrimination accuracy of this threshold to detect LN involvement as of 86%, 85% and 86%, respectively.

Finally, image-guided sLND can be considered as a feasible and useful treatment option in patients with clinical LN failure after primary defnitive treatment such RARP.

*Vipul Patel, MD and Vladimir Mouraviev, MD* *Director of Urologic Oncology* *Florida Hospital Cancer Institute, FL, USA* *E-mail: vipul.patel.md@flhosp.org*

## References

[1] Abdollah F, Briganti A, Montorsi F, Stenzl A, Stief C, Tombal B (2015). Contemporary role of salvage lymphadenectomy in patients with recurrence following radical prostatectomy. Eur Urol.

[2] Kabasakal L, Demirci E, Ocak M, Akyel R, Nematyazar J, Aygun A (2015). Evaluation of PSMA PET/CT imaging using a 68Ga-HBED-CC ligand in patients with prostate cancer and the value of early pelvic imaging. Nucl Med Commun.

[3] Eiber M, Beer AJ, Holzapfel K, Tauber R, Ganter C, Weirich G (2010). Preliminary results for characterization of pelvic lymph nodes in patients with prostate cancer by diffusion-weighted MR-imaging. Invest Radiol.
